# Signaling Involved in Hair Follicle Morphogenesis and Development

**DOI:** 10.3390/ijms15011647

**Published:** 2014-01-22

**Authors:** Pisal Rishikaysh, Kapil Dev, Daniel Diaz, Wasay Mohiuddin Shaikh Qureshi, Stanislav Filip, Jaroslav Mokry

**Affiliations:** 1 Department of Histology and Embryology, Medical Faculty in Hradec Kralove, Charles University in Prague, Simkova 870, 500 38 Hradec Kralove, Czech Republic; E-Mails: rishikaysh2007@gmail.com (P.R.); kapildchauhan@rediffmail.com (K.D.); danieldiazg@gmail.com (D.D.); wasay9710@yahoo.com (W.M.S.Q.); 2 Department of Oncology and Radiotherapy, Medical Faculty in Hradec Kralove, Charles University in Prague, Simkova 870, 500 38 Hradec Kralove, Czech Republic; E-Mail: filip@fnhk.cz

**Keywords:** hair follicle, morphogenesis, stem cell, signaling, Wnt, Shh, Notch, BMP

## Abstract

Hair follicle morphogenesis depends on Wnt, Shh, Notch, BMP and other signaling pathways interplay between epithelial and mesenchymal cells. The Wnt pathway plays an essential role during hair follicle induction, Shh is involved in morphogenesis and late stage differentiation, Notch signaling determines stem cell fate while BMP is involved in cellular differentiation. The Wnt pathway is considered to be the master regulator during hair follicle morphogenesis. Wnt signaling proceeds through EDA/EDAR/NF-κB signaling. NF-κB regulates the Wnt pathway and acts as a signal mediator by upregulating the expression of Shh ligand. Signal crosstalk between epithelial and mesenchymal cells takes place mainly through primary cilia. Primary cilia formation is initiated with epithelial laminin-511 interaction with dermal β-1 integrin, which also upregulates expression of downstream effectors of Shh pathway in dermal lineage. PDGF signal transduction essential for crosstalk is mediated through epithelial PDGF-A and PDGFRα expressed on the primary cilia. Dermal Shh and PDGF signaling up-regulates dermal noggin expression; noggin is a potent inhibitor of BMP signaling which helps in counteracting BMP mediated β-catenin inhibition. This interplay of signaling between the epithelial and dermal lineage helps in epithelial Shh signal amplification. The dermal Wnt pathway helps in upregulation of epithelial Notch expression. Dysregulation of these pathways leads to certain abnormalities and in some cases even tumor outgrowth.

## 1. Introduction

The hair follicle (HF) is considered a mini-organ formed with neuroectodermal-mesodermal interaction [1]. HF morphogenesis starts at an early embryonic stage. Its proper development and regular cycles involves a strong interplay between Wnt, Hedgehog, Notch and bone morphogenetic protein (BMP) signaling pathways. The stages of morphogenesis are broadly classified into: induction, organogenesis and cytodifferentiation. During the induction stage, Wnt mediated signal transduction first arises in mesenchymal cells directing the thickening of overlying epithelial cells to form a placode. The organogenesis stage consists of complex interplay of signals; epithelial cells direct the underlying dermal cells to proliferate and form a dermal condensate, which in turn signals the epithelial cells to proliferate and grow downwards into the dermis. In cytodifferentiation, the dermal condensate is enveloped with follicular epithelial cells thus forming distinct dermal papilla, which instruct the ectoderm to shape the entire HF through the action of morphogens and growth factors [1]. Proper positioning and spacing of HF (*i.e*., primary HF pattern) is mediated via the ectodysplasin receptor Edar-BMP signaling and transcriptional interactions. Edar-directed stabilization of β-catenin is important in determining the position of the follicle [2].

HF undergoes characteristic cyclic phases known as the hair cycle. The cycle includes anagen (growth phase), catagen (regression phase) and telogen (resting phase); exogen (release of the telogen club or hair shredding) does not occur at every cycle [3]. Anagen or growth phase determines the length of the hair shaft by means of inner root sheath (IRS) and hair shaft, which together contributes to seven layers [3]. Catagen features a substantial decrease in cell cycling because of increased apoptosis in epithelial cells of bulb, outer root sheath and outermost epithelial layer. Club shaped bulb indicates attainment of the hair shaft (HS) differentiation; finally, catagen is followed by a resting phase known as telogen [3]. Molecules that promote the induction of catagen have been identified as FGF5, BDNF, p75, p53, TGFβ1 and BMPRIa [1,4–7]. Telogen to anagen transition is dependent upon many factors and since during the telogen phase the HF strongly expresses estrogen receptors, the binding of 17-β-estradiol to these receptors prevents HFs from exiting the telogen phase to enter anagen phase [8].

## 2. Signaling and Stem Cell Niche

The stem cell microenvironment “niche” [9] plays a crucial role in cell fate decision by controlling self-renewal and differentiation. Architectural organization of molecules and factors are inevitable for proper HF development and maintenance. For instance, loss of a critical transcription factor LHX2 from the niche results in HF cellular disorganization and transformation into a sebaceous gland [10]. The Wnt signaling pathway plays an important role in the maintenance and proliferation of stem cell reservoirs [11]; dysregulation of this pathway often results in the development of familial and/or sporadic epithelial cancers [11].

**Figure 1.**
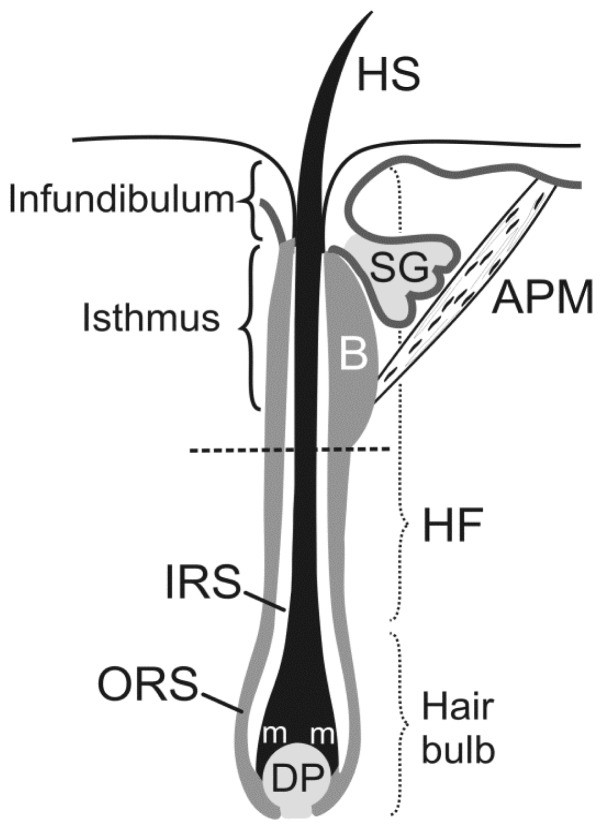
Hair anatomy. The hair root comprises a hair follicle (HF), a hair bulb, and a dermal papilla (DP). The outer root sheath (ORS) is a direct continuation of the Malpighian layer of the epidermis. The bulge (B) is located at the insertion site of the arrector pili muscle (APM) into the ORS. The insertion of the sebaceous gland (SG) duct forms the anatomical interface between the infundibulum and isthmus. A dotted line separates an upper transient portion of the hair from a lower permanent portion. IRS, inner root sheath; m, matrix; HS, hair shaft.

In adult HFs (Figure 1), the most primitive epidermal stem cells are restricted to the central isthmus directly above the bulge [12]; these cells express LGR6 and are Wnt-independent. During development, LGR6^+^ cells are located in hair placodes and prenatally they give rise to HFs, sebaceous glands and interfollicular epidermis. Postnatally, their contribution to hair lineages diminish; they renew epidermal cells and sebocytes and give rise to LGR5^+^ stem cells that are Wnt-dependent and contribute in HF lineages. Nevertheless, following wounding, LGR6^+^ cells contribute to the epidermis healing including formation of new HFs. Hierarchically lower epithelial stem cells are dependent on Wnt signaling due to the presence of LGR4 and LGR5, an orphan group of G protein coupled receptors. These receptors express high affinity toward R-spondins and their binding causes increased Wnt/β-catenin signaling by enhanced Wnt-induced LRP6 phosphorylation [13]. HF morphogenesis is regulated by SGK3 and Akt2 in postnatal HF through modulation of β-catenin dependent transcription processes [14]. Dlx3 is a homeobox transcription factor essential for hair morphogenesis, differentiation and cycling programs. Dlx3 is a direct target of Lef1 regulation as it is placed downstream of Wnt and its repression causes loss of BMP signaling. Loss of Dlx3 leads to enhanced proliferation and delayed regression such as in Tricho-Dento-Osseous syndrome caused by Dlx3 mutations [15].

There is strong genetic evidence showing that progenitor cells at the bottom of the intestinal crypt accumulate nuclear β-catenin, a hallmark of active Wnt signaling [16], therefore playing a critical role in the regulation of epithelial stem cells in the intestinal tract [17,18]; similar evidence has also been found in HF [19]. Abrogation of Wnt signaling by removal of either Tcf4, β-catenin or by overexpression of Wnt inhibitor Dickkopf 1 (Dkk-1) results in complete loss of cell proliferation [18,20,21]. Wnt reporter activity starts with the binding of the Wnt ligand with the Frizzled receptor, making the scaffold unable to function, resulting in the accumulation of β-catenin in the cytoplasm. Starting the signaling cascade, Tcf/Lef interacts with DNA-binding proteins to which stabilized cytoplasmic β-catenin can bind, acting as a transcriptional co-factor for their target genes. Tcf4 is prominently expressed in the stem cell compartment [22]; in the follicular stem cell niche, Tcf4 is expressed in conjunction with Tcf3 [23]. Mice deficient for Lef1 do not develop HFs [24]. Separation of β-catenin results in an absence of follicle morphogenesis in the neonate [25] and a postnatal loss of the follicle stem cell niche [26]. In the same manner, the overexpression of Dkk1 results in failure to develop HF [27], which provides further evidence for the specific role of Wnt signaling during the development and maintenance of epithelial stem cells.

During telogen bulge stem cells reside in a Wnt-restricted environment [28]. During the transition between hair cycle telogen and anagen phases, nuclear β-catenin and Lef1/β-catenin reporter expression can be detected at the bulge base, where the new HF begins to emerge [27–29]. Consistent with the role of Wnt signaling in stem cell activation, transgenic mice expressing a stabilized form of β-catenin exhibit *de novo* formation of HF and increased follicle density [30] as well as precocious re-entry into the regenerative phase of hair growth [27,31,32]. In the HF, strengthening of β-catenin activates bulge stem cell proliferation and regeneration of HF. Through transcriptional profiling of purified bulge stem cells during telogen and anagen phases of the hair cycle, it was discovered that during stem cell activation a number of genes associated with cell cycle progression are expressed. However, Wnt signaling in bulge stem cells did not upregulate the hair keratin genes which are induced at later stages of HF differentiation [27].

Wnt signaling is not the only signal transduction pathway that instructs stem cells. Notch signaling controls selective cell-fate determination in a variety of tissues [33]. Notch signaling plays an important role in HF development and it has been suggested that it is also required for follicular fate selection of adult HF stem cells in the bulge and contributes to the maintenance of the follicular structure but not to cell fate selection during HF morphogenesis [34]. In addition, Notch signaling ensures an optimal matrix proliferating environment during the first anagen by suppressing TGF-β and activating the Kit ligand [35]. In the anagen phase of HF bulb, three Notch receptors are expressed in partially overlapping domains [34]; each follicle is derived from two to four multipotent bulge stem cells [36,37] which give rise to oligo-lineage HF progenitors [38] that are located adjacent to the dermal papilla in the matrix. In contrast to Wnt signaling [25], neither arm of Notch signaling is required for follicular fate selection by bulge stem cells. Instead, Rbpj-dependent Notch signals restrict bulge cells (or their uncommitted, migratory descendents) to the follicular fate. In addition to the selection of a follicular fate, a substantial fraction of Notch/Rbpj-deficient stem cells spawn progeny able to spontaneously choose an epidermal fate and migrate upwards, joining the interfollicular epidermis and producing epidermal cells deficient in terminal differentiation. HFs formed by Notch-deficient stem cells are associated with dermal papillae, producing a bulb expressing hair keratins but failing to maintain the identity of IRS cells and medulla [34]. In the absence of Notch signaling, bulge stem cell descendants retain their capacity to execute the follicular differentiation program but fail to maintain it owing to their genetic deficiency.

BMPs are secreted signaling molecules that belong to the TGF-β superfamily and exert their biological activity via interaction with specific BMP receptors [39–42]. In the extracellular space, BMP activity is modulated by BMP antagonists that regulate the magnitude and spatio-temporal specificity of signaling through BMP receptors [43,44]. BMPs act as multifunctional regulators of vertebrate development, controlling cell proliferation, differentiation, and apoptosis in various organs including the skin [45–47]. In postnatal life, BMPs also play important roles in normal tissue remodeling and homeostasis [45,47–49]. BMPs interact with members of other growth factor families (Wnt, Shh, TGF-β, EGF, FGF, Notch, neurotrophins) to control cell proliferation, differentiation, and apoptosis in the developing skin and its appendages.

Although the mechanisms controlling the hair cycle are still unfolding, BMP signaling is likely to be involved. BMP signaling also plays a role in HF morphogenesis, postnatal regeneration and control of the HF cycle through regulation of hair matrix precursor cell proliferation and differentiation [47]. Enhanced BMP signal activation by ectopically expressing BMP4 or targeted inactivation of the BMP antagonist Noggin [50] results in significant retardation of HF induction and progressive baldness. Interaction with exogenous BMPs stimulates the transmembrane receptor BMPR1A to phosphorylate Smad 1, 5 and 8 that signal in trimeric complexes with Smad4. Noggin, an extracellular BMP inhibitor, is expressed by mesenchyme, where it induces follicle morphogenesis in the embryo and promotes new HF growth (anagen) postnatally [50,51]. Interestingly, once embryonic HFs have been initiated, they express BMP4, suggesting a negative feedback loop to prevent new HF initiation in the vicinity. In adult follicle stem cells, Smad1 is phosphorylated and BMP6 levels are elevated, suggesting that BMP signaling is active in the bulge [4,23]. Conversely, in the early hair germ that emerges from the activated bulge, nuclear P-Smad1 is diminished and remains low in the developing outer root sheath (ORS) and in the lower part of the matrix. The strongest BMP signaling is in the cells that differentiate to produce the IRS and hair shaft [52]. In normal HFs, the *Sox4* and *Sonic hedgehog* (*Shh*) genes are not expressed in the bulge but are co-expressed in the developing hair germ [27,53]. HF stem cells display signs of activated TGF-β and BMP signaling *in vivo*, and *in vitro*, ligands specific for each pathway cause proliferating keratinocytes to transiently withdraw from the cell cycle [23,54–56]. Thus, it was surprising that ablation of BMP signaling alone was sufficient to disrupt the quiescent state of the HF stem cell niche. In the absence of BMPR1A, quiescent stem cells were precociously activated to enter the proliferative phases associated with the new hair cycle. BMP6 and BMP4 seem to play autocrine and paracrine roles, respectively, in niche quiescence [23]. Conversely, BMP antagonists, including Noggin, Gremlin, and ecto-dysplasin, are made by the dermal papilla (DP) and, hence, are likely to play paracrine roles in stem cell activation [47,57]. Sox9, Lhx2, Sox4, and Shh, which are characteristic of quiescent bulge cells and/or their early proliferating progeny, were expanded after inhibiting BMP signaling. The importance of β-catenin stabilization and activation of Lef/Tcf target genes in follicle formation is well established [25,30]. This process involves signaling by ectodermal Wnts, which when blocked by overexpression of Dkk1, suppress follicle formation [45]. The process also involves inhibition of BMP signaling, given that Lef1 and follicle formation are repressed in the absence of Noggin. Additionally, Lef1/β-catenin-mediated transcription is lost in *Noggin*-null mutants, and is elevated in the presence of excessive Noggin or in the absence of BMPR1A [51,52].

Sonic Hedgehog (Shh) signaling regulates proliferation and developmental patterning in many tissues, including the HF [58,59]. Shh is crucial for hair development and cycling, and the deregulated function of members of the Shh signaling cascade alters HF formation and generates epidermal neoplasia [60]. In addition to maintaining the cycling follicle, bulge cells can also be recruited to the interfollicular epidermis after wounding [61]. Keratin 15 expressing bulge cells migrate to the healing epidermis during re-epithelization, but they do not persist in the regenerated epidermis [62]. Hh signaling has been proposed to regulate both stem cell maintenance and, at higher signaling levels, cell proliferation in many adult epithelia [63]. In anagen skin, Shh is expressed at high levels in the follicle matrix and acts as a mitogen that drives anagen regeneration [30,64]. Shh signaling is also essential for proper control of HF morphogenesis [65], as well as involved in controlling the hair cycle initiation in both primary and secondary HFs [66–68].

## 3. Hair Follicle Morphogenesis

**Figure 2.**
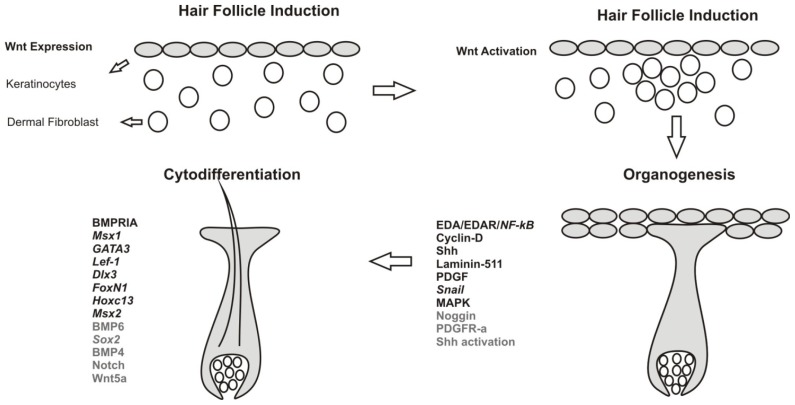
Stages of hair follicle morphogenesis. HFs are formed by interaction between epithelium (keratinocytes) and underlying dermal fibroblasts. Comprehensive lists of signaling molecules (capitals) and transcription factors (italics) are provided for each stage. Signaling molecules and pathways indicated in black are either expressed or activated in keratinocytes while grey ones are associated with dermal fibroblasts.

HF morphogenesis is classified into three main stages: induction, organogenesis and cytodifferentiation (Figure 2). Regulatory roles of Wnt, Shh, Notch, Bmp and other accessory pathways in each of these stages are discussed below.

### 3.1. Hair Follicle Induction

HF development is an orchestrated interaction between mesenchymal and epithelial cells mediated through the secretion of signaling molecules [69]. Wnt is the first signal essential for HF induction. Specific ligands contributing to first signal are still not delineated while Wnt5a mediates a “second dermal signal” required for HF proliferation downstream from Shh expression in the overlying epithelium [70]. Wnt secretion is mediated by Wntless (Wls), which is a trans-membrane cargo protein. Its role in HF induction is obscure but it is expressed in developing placodes, embryonic epidermis and HF compartments after completion of hair morphogenesis [71]. As per current evidence Wnt can be classified as primary Wnts (Wnts 3, 4 and 6) and secondary Wnts (Wnts 2, 7b, 10a and 10b). Primary Wnts are essential for HF initiation while secondary are involved in HF development [27,72]. Epidermal Wnt ligands are essential for stabilizing and regulating dermal β-catenin signaling and fibroblast proliferation. Absence of dermal β-catenin signaling is characterized by down-regulation of epidermal β-catenin activity and Edar expression. This proves that dermal β-catenin signaling is indispensable for fibroblast proliferation and HF induction. During HF induction dermal fibroblast aggregation is an essential step. This fibroblast aggregation is directly linked to Wnt/β-catenin signaling, which is mediated by regulating versican expression [73].

Stabilized dermal β-catenin activity is essential for expression and relaying signals to the overlying epithelium; the nature of this signaling is still elusive. β-catenin expression is sequentially activated starting first in upper dermis followed by epithelium of hair placodes [72,74] and at last in dermal condensates [28,75]. Dermal signals direct epithelial thickening known as HF placodes [76] and hair placodes in turn direct clustering of underlying fibroblast [1].

Epithelial Wnt/β-catenin and EDA/EDAR/NF-κB signaling pathways play role in HF initiation and primary hair placodes maintenance. Wnt/β-catenin regulates expression of Eda [77,78] as well as expression of its receptor Edar [2,57]. Edar signaling has been implicated in refining Wnt/β-catenin pattern during primary placodes induction and it also acts as a suppressor of BMP signals, which has an inhibitory effect on placode formation [2,57]. In the absence of NF-κB or Edar signaling the placode borders are irregular and these patches of cells appear to be fused or string shaped. NF-κB plays a characteristic role not only in refining pattern of placode borders but also in further development of the placode. The mechanisms involved behind these functions are very intriguing and involve the expression of Wnt signaling inhibitor Dkk4, and Wnt ligands Wnt10b and Wnt10a. Expression of Dkk4 is regulated by NF-κB as well as by LEF/TCF/β-catenin. NF-κB refines placode borders by indirectly modulating Wnt signaling. Wnt10b is a direct target of the Edar pathway. NF-κB is essential for maintaining Wnt10b and Wnt10a levels [72]. Precise function of Wnt10b in HF development is still unknown. Competition between placode promoting signals (Wnt10b and β-catenin) and inhibitory signals (Dkk4) is essential for establishing the regular array of placodes [79].

Placode formation is followed by dermal fibroblasts condensation. Condensation is mediated by fibroblast growth factor (FGF) 20 [80] which is expressed in hair placodes and is induced by epithelial Eda/Edar and Wnt/β-catenin signaling to facilitate underlying dermal condensation. FGF20 governs formation of primary and secondary condensation in developing HFs. In order to mediate FGF20 signaling, corresponding receptors must be present in the dermal fibroblasts, but to date no specific receptor has been identified. FGFR1 is a good candidate as it is expressed in the upper dermis at the time of HF induction [81]. FGF signals function during multiple stages of HF development. FGFR2B is essential for HF development while its deletion causes retarded HF development [82,83]. FGF7 functions as an inhibitor of HF induction [84].

HF initiation in placodes requires down regulation of keratinocyte growth factor (KGF) and epidermal growth factor (EGF) signaling. Receptors of these pathways are down regulated (EGFR and FGFR IIIb) while their ligands persist throughout the initiation. In the presence of their ligands HF formation is inhibited in a time and dose dependent manner. The main function of these pathways is to promote epidermal differentiation at the expense of HF fate [83]; however, details concerning these pathways are yet to be obtained.

### 3.2. Hair Follicle Organogenesis

Placode proliferation and dermal condensation occur concomitantly and massive keratinocyte proliferation marks the beginning of organogenesis. In order for HF induction to proceed, it is essential to antagonise the inhibitory effect of BMP. Dermal Noggin mediates inhibition of BMP and regulates HF epithelial induction through Lef1 [50,51]. Sonic hedgehog (Shh) expression is a crucial HF inductive signal generated in the HF placode [85,86]. EDA/EDAR/NF-κB is a crucial signaling pathway for progression of organogenesis. Ectodysplasin-A(EDA)/EDAR/NF-κB signaling is implicated in the activation of epithelial Shh and cyclin D1 expression [87]. The EDA/EDAR signaling pathway can be briefly summarized as EDA → EDAR → EDARADD → NF-κB → Shh → Cyclin D1. Cyclin D1 expression is induced by Shh- or Wnt10b-signaling [1,27,78], and the role of Shh in its expression is indispensable [88]. Shh signaling is dispensable for HF induction but it is required for epithelial proliferation and hair placode down growth [65,88,89]. Shh induces Shh signaling in both the compartments of the HF *i.e*., epithelium and dermal condensate. The initial role of Shh signaling in early HF is epithelial proliferation [90–93]. Epithelial Shh regulates DP maturation and maintains DP functions via Noggin, which is essential to drive HF morphogenesis. Shh expression is regulated by Wnt signaling through Lef1-mediated downregulation of E-cadherin, which in turn increases Shh levels [65]. In the primary hair placode, epithelial Wnt signaling is inhibited by BMP. Dermal Noggin secretion is essential to rescue BMP mediated inhibition and recent evidence shows that sustained Shh expression relies on BMP inhibition mediated by dermal Noggin [19]. Shh activation of Noggin involves a rather complex set of events. Epithelial-mesenchymal crosstalk is essential for Noggin expression as well as for other signal exchange. Noggin expression relies on epithelial Shh expression, epithelial laminin-511, epithelial platelet-derived growth factor (PDGF), dermal β intergrin and dermal expression of PDGFRα. Epithelial derived laminin-511 interacts with mesenchymal β integrin promoting primary cilia formation, which in turn mediates epithelial derived Shh to initiate signaling through activation of downstream Shh effectors, like patched, smoothened and Gli. Epithelial PDGF activates mesenchymal PDGFRα, and this, combined with Shh signaling activates Noggin secretion by mesenchymal (dermal) cells. Noggin inhibits BMP signaling in epithelial cells, which leads to Lef1 expression thereby rescuing stalled epithelial Wnt signaling. Thus, laminin-511 amplifies both mesenchymal Shh signal by primary cilia formation and epithelial Shh expression by Noggin-mediated BMP inhibition [94].

HF morphogenesis stalls in the absence of TGF-β2 signaling and Snail expression (involved in expression of certain adhesion proteins) by blocking HF down growth. TGF-β2 signaling is essential for transient induction of transcription factor Snail and activation of Ras-mitogen-activated protein kinase (MAPK) pathway in the bud. Snail functions in regulating cell proliferation and cell adhesion. Therefore this pathway precisely governs epithelial proliferation, junctional remodeling and bud formation which helps in proper progression of hair morphogenesis [95].

### 3.3. Cytodifferentiation

HF differentiation is characterized by development of all the compartments of the HF. Various signaling molecules elicit their role in closely refining this process. IRS differentiation is regulated by the Gata3 and Cutl transcription factors [96–98], while BMP signaling and transcription factors such as Msx2, FoxN1 and Hoxc13 regulate hair shaft differentiation [99–104].

Wnt signaling induces a positive effect on the Notch pathway while recent evidence has shown an antagonistic effect of Notch on Wnt signaling [105,106]. In dermal papillae, the Notch/RBP-Jk signaling pathway activates Wnt5a expression facilitated by binding of Notch1 to the RBP-Jk binding site on the promoter region. Wnt5a mediates Notch signaling by facilitating expression of the *FoxN1* gene. FoxN1 plays an important role not only in regulating HF keratinocyte differentiation [107] but also in signaling specific transfer of pigment from melanocytes to keratinocytes of the hair cortex [108]. HF differentiation is regulated by the underlying mesenchymal through Notch-CSl pathway, with Wnt5a and FoxN1 mediators [109].

The *Notch* gene family (*Notch1*, *Notch2*, *Notch3*) comprises highly conserved transmembrane receptors. Notch participates in HF development in three possible ways: lateral inhibition, boundary formation and lineage decision. Cell fate is dependent on asymmetrical inheritance of Notch regulators and regulating differentiation and promoting boundary formation by altering adhesive properties of keratinocytes [110], *i.e*., it plays a role in control of cell fate, stem cell potential and differentiation [111,112]. Notch induces differentiation by suppressing p63 expression [113]. Notch, Wnt and vitamin A are part of interconnected pathways that play roles in epidermal lineage selection [114]. By modulating cell adhesion properties Notch determines cellular location and thus in turn regulates differentiation, mainly in the epidermis [115].

Hair shaft progenitor differentiation is under direct control of DP through Sox2 expression. BMP6 and Sostdc1 are direct transcriptional targets of Sox2. Sox2 leads to concomitant upregulation and downregulation of BMP6 and Sostdc1, respectively. Sostdc1 is a potent BMP inhibitor. BMP6 inhibits cell migration. Thus, Sox2 regulates hair growth by controlling progenitor cell migration through BMP-mediated epithelial-mesenchymal crosstalk [116].

BMP and BMPRIA have been implicated in HF differentiation [52]. Along with its classical function in epithelial stem cell maintenance and progenitor differentiation it has recently been implicated in DP signature cell maintenance, which plays a crucial role in epithelial-mesenchymal crosstalk [117]. BMP mediates its effect through its receptor BMPRIA, which is the only known BMP receptor expressed in HFs [50]. BMPRIA is essential for differentiation of progenitor cells of the inner root sheath and hair shaft. BMP4 induces GATA3, which acts as terminal differentiation factor [118]. GATA3 in turn maintains BMP levels thus establishing a feedback loop [119,120]. Thus BMPRIA activation promotes IRS progenitor cells differentiation through GATA3 [52]. Wnt signaling regulates hair shaft differentiation and this function is mediated through BMPRIA signaling. Sequential inhibition and activation of BMPRIA in progenitor cells maintains enough Lef1 and stabilized β-catenin to activate the HF specific keratin and generate hair shaft [52]. How BMP is activated in later stages of development is still unclear. Recent evidence suggests that initially active epithelial BMP4 activates Msx1 and several other transcription factors, which in turn induce dermal or mesenchymal BMP4 expression [121].

Other factors controlling differentiation include the Dlx3 transcription factor that controls differentiation of IRS and hair shaft. Dlx3 is a direct target of Lef1 and upregulates expression of Hoxc13 and Gata3 transcription factors, potent regulators of hair shaft differentiation [15].

## 4. Hair Follicle Regeneration

The process of HF regeneration involves BMP antagonism and subsequent activation of Wnt and other underlying pathways. The initial steps of regeneration involve crosstalk between quiescent epithelial stem cells and mesenchymal dermal papilla forming a repressive environment for BMP signals.

TGF-β2 is an important factor essential for regeneration as it activates the Smad2/3 pathway in HF stem cells, which is crucial for avoiding delayed regeneration. *Tmeff1*, a target gene of the TGF-β2/Smad2/3 pathway, lowers the threshold of BMP in the niche promoting stem cell transition from telogen to anagen [122]. Recent evidence suggests that stem cells involved in HF formation have to transit through each phase of the hair cycle and remain in each phase for an appropriate time period; failure to do so eventually leads to stem cell exhaustion. In order to prevent this from happening, the factor known as MED1 enables the quiescent state maintenance of HF stem cells, thus assisting in the normal hair cycle progression [123].

Normally, the hair cycle undergoes three phases named anagen, telogen and catagen [19,85,124]. During quiescence, the HF stem cells reside in the bulge [125]; within this niche, bulge stem cells surround the hair shaft that is developed in the previous cycle. Telogen to anagen transition is facilitated by DP-HF stem cell crosstalk and the interplay between Wnt and BMP inhibitory factors [101,124,126–129]. During regeneration, hair germ stem cells are the first to be activated later followed by bulge stem cells [124]. During anagen, the DP delimits the newly formed hair bulb and stimulates the undifferentiated bulge cell progeny, which are present along ORS, to proliferate. This proliferation leads to the formation of transiently amplifying matrix cells that undergo a few divisions while in contact with DP and then terminally differentiate to form the hair and IRS. At the point of anagen to catagen transition, matrix cells undergo apoptosis and the DP retracts upward along the epithelial strand. EGF and EGFR promote the catagen and IGF-1 promotes the anagen via MAPK and phosphatase and tensin homolog (PTEN). The outcome of growth factor signaling is dependent on the cell type, the signal received, and the receptor. Anagen HF consists of multiple growth factors for tyrosine kinase receptors including insulin-like growth factors (IGFs), EGF, FGFs, and PDGF [130]. The two principle signaling routes that are activated by growth factor receptor tyrosine kinases are the Ras–Raf–MEK-ERK (extracellular regulated kinase) [131] and phosphatidylinositol 3 kinase (PI3K)–PDK1–Akt pathways [132]. The RAS pathway regulates cellular migration, proliferation, survival, differentiation, and senescence by stimulating various parallel effector pathways [133,134]. Mutations in *RAS* gives rise to genetic disorders termed RAS/MAPK syndromes. Mutations in this gene affect HF morphogenesis, differentiation and hair cycling. RAS signaling mediates these effects by repression of Shh, and is supposed to be a mediator of FGF signaling. RAS signaling regulates HF morphogenesis by fine-tuning Shh levels [135]. At the onset of telogen, BMP signals from the inner layer of non-stem cells niche cells [136] and from surrounding dermal tissue impose a threshold that must be overcome to initiate the next cycle and then DP is dislodged from the niche in order to maintain quiescence [136].

Nuclear factor 1 C (NF1C) is a key regulator in the transition from telogen to anagen phase in the hair cycle by inducing the expression of Shh, Wnt5a and Lef1. It also increases keratinocyte proliferation and initiates the induction of anagen by upregulating TGFβ1 and the inhibition of p21. The function of this transcription factor is required for postnatal tissue regeneration and adult progenitor cell proliferation [137].

During embryonic HF formation the RunX1 transcription factor regulates HF stem cells emergence and maintenance via modulation of bidirectional cross talk between nascent stem cells and their niche. Detailed analysis revealed embryonic mesenchymal RunX1 expression is essential for proper adult HF stem cell differentiation and long-term skin integrity. In its absence, HF stem cells differentiate into sebaceous glands [138]. RunX1 function is dispensible during embryonic HF induction but it is essential for regeneration. Controlling stem cell quiescence is essential for normal homeostasis and for preventing cancer. In HF stem cells, RunX1 promotes proliferation and simultaneously represses *p21*, *p27*, *p57*, and *p15* transcription. Out of these genes, p21 acts as cell cycle inhibitor. Runx1 and p21 synergistically rescue cell cycle arrest depending on the required conditions by directly down-regulating mRNA levels of other cyclin-dependent kinase inhibitors [139].

miR-31 plays a significant role during HF growth and hair fiber formation by targeting a number of growth regulatory molecules and cytoskeletal proteins. Its expression increases during anagen and subsides during catagen and telogen phases. miR31 negatively regulates expression of *Fgf10*, sclerostin, and BMP and activin membrane-bound inhibitor homolog (*BAMBI*), *Dlx3* and a few keratin genes. Out of these genes, sclerostin and *BAMBI* are components of Wnt signaling while *Dlx3* is of BMP signaling. Out of these arrays of genes, *Krt16*, *Krt17*, *Dlx3*, and *Fgf10* serve as direct miR-31 targets. Thus miR31 regulates HF growth by modulating growth-regulating molecules and cytoskeletal proteins [140].

## 5. Disorders Associated with Hair Follicles

HF disorders can be classified mainly into three types: alopecia, hirsutism and hair shaft disorders. Alopecia is characterized by abnormal loss or thinning of hairs. A common disorder associated with HF includes androgenic alopecia caused due to hormonal imbalance. Alopecia, hirsutism (excessive hair growth in adult women) and hair shaft disorders are mainly caused not only by the malfunction of one or more signaling pathways but also by other factors such as autoimmune disorders, genetic predisposition, cancer and microbial infections.

### 5.1. Alopecia

Various forms of alopecia involve deviations from normal signaling pathways, for instance hypotrichosis simplex disease, which is an autosomal disorder characterized by miniaturization of HFs [141]. *Adenomatosis polyposis down regulated 1* (*APCDD1*) gene encodes a membrane bound glycoprotein that acts as a potent inhibitor of the Wnt signaling pathway by interacting with Wnt3A and LRP5. This inhibitory function plays an important role during development of neurons from the progenitors; however, L9R mutations inhibit membrane localization and stability thus rendering it free to act within the Wnt signaling pathway, predisposing the patient to alopecia [142]. Trichorhinophalangeal syndrome is characterized by scarce scalp hair in association with other abnormalities; mutations in zinc finger transcription factor Trps1 is considered to be the cause of this disorder since Trps1 is considered essential for normal HF development [143].

One key factor in non-androgenic male pattern baldness is Dickkopf1 (DKK-1), which promotes catagen. It is suggested that DKK-1 blocks Wnt signaling by preventing β-catenin activation and instead activating the pro-apoptotic protein Bax, inducing apoptosis in outer root sheath keratinocytes. Current research supports that DKK-1 causes anagen to catagen induction leading to pattern baldness, which is commonly shown by older male populations [144].

The main cause of male pattern baldness in response to hormones or androgenic alopecia is due to dihydrotesterone, which is produced by balding dermal papillae. Presence of dihydrotesterone in HF occurs either through the DP capillaries, as it is normally circulated in the blood, or by conversion of testosterone in balding DP cells. Presence of dihydrotesterone causes upregulation of IL-6 that in turn promotes the expression of the IL-6 receptor along with glycoprotein 130 in keratinocytes and matrix cells. The outcome of this IL-6-upregulated expression is inhibition of the hair shaft elongation with simultaneous suppression of matrix cells proliferation. Studies in this regard have shown that IL-6 expression induces anagen-catagen progression [145].

Shh pathway is known to play an important role in both embryonic hair development as well as in the normal cycling of adult HF, and implies in the induction of anagen phase accompanied by hair growth [82]. Certain defects related to catagen HF arrest can be treated with the ectopic activation of Shh pathway that promotes catagen to anagen transition, restoring normal hair growth. However, caution must be taken, as excessive Shh activation can lead to skin cancer.

A few miRNAs have been implicated in male patterned baldness. Recent evidence has demonstrated upregulation of miR-221, miR-125b, miR-106a and miR-410 in balding papilla cells [146]. miRNAs have an established role in the functioning of androgenic receptors [147]. Androgen secretion affects hair color and size via the hair growth cycle. miR-221 targets functioning of receptor tyrosine kinase, c-kit, and the cyclin-dependent kinase inhibitors p27/kip1 and p57/kip2 [148]. miR-221 may impede SCF/c-kit signaling, which is involved in melanocyte migration and maintaining pigmentation. To date, no specific role has been assigned to these miRNAs, and further research is necessary to evaluate their upregulation in alopecia or male patterned baldness.

### 5.2. Hair Follicle Tumors

HF associated tumors are rare and often associated with hair germ, infundibulum, ORS and matrix or follicle mesenchyme. Generally HF tumors arise due to unrestrained signaling pathways. Examples of HF tumors include trichofolliculoma, trichoepithelioma, trichoblastoma, trichilemmoma, and trichomatricoma *etc*.

Notch and BMP pathways are proliferation antagonists and suppress epithelial growth. Loss or mutation of these factors can facilitate tumor formation as evidenced from inhibition of BMP signaling in hair progenitor cells that leads to trichofolliculoma in humans [149].

The Wnt pathway, along with its downstream effector β-catenin, plays an important role in cell proliferation, epithelial architecture and cell polarity regulation. The stabilization of β-catenin is essential for HF development but its dysregulation leads to HF tumors, e.g., trichomatricoma [150]. Additionally, a non-canonical Wnt ligand is linked with squamous cell carcinoma and basal cell carcinoma, which are invasive skin tumors.

The Wnt pathway effector β-catenin activates downstream target genes either independently or in synergy with other factors and response elements. One of these response elements, vitamin D, is essential for β-catenin induced hair differentiation without which β-catenin is unable to carry out its physiological effect. Recent evidence has shown that β-catenin and vitamin D response elements are highly upregulated in human trichofolliculomas [151].

Basal cell carcinoma is also linked with Shh pathway dysregulation. Evidence has strongly suggested an association between Shh and the insulin like growth factor binding protein-2 (IGFBP-2) in the transformation of normal HF to basal cell carcinoma [152]. The origin of basal cell carcinoma is still unknown; although an X-ray tracking assay has confirmed follicular bulge stem cells expressing keratin 15 being responsible for basal cell carcinoma [153]. Injury or wound promotes the inflow of cells for regeneration; in some cases, this infiltration of cells leads to epithelial tumors. In depth analysis pinpoint mutations in smoothened (Smo) as a possible reason for epithelial tumors. For instance, during the healing process, if cells carrying Smo mutations are recruited to the injured site, they will trigger unregulated downstream Shh pathway causing superficial basal cell carcinoma like tumors [154]. The effects of Shh are regulated by the degradation of the transcription factor Gli1. Accumulation of Gli1 leads to different types of cancer including basal cell carcinoma. Usually, Gli1 contains two independent degradation signals D (N) and D (C). Loss or removal of these signals stabilizes Gli1 protein thus promoting tumor formation [93].

Some very intriguing mechanisms underlie tumor cell formation given that they are massively reprogrammed back to embryonic HF cell progenitors. The expression of β-catenin is highly upregulated followed by the expression of SmoM2 in adult epidermis; deletion of β-catenin prevents progenitor reprogramming and tumor formation [155]. This proves that, as the interlinking of pathways are essential for normal HF induction and development, their dysregulation or mutation could also lead to cancer. Pathway interlinking in tumor formation is still not well understood, although recent evidence has described the role of mammalian target of rapamycin (mTOR) in linking different signaling pathways together in trichofollicular tumorigenesis [156].

BMP plays an essential role in controlling various developmental processes and acts as a potent tumor suppressor. Recent evidence has revealed a role of miRNA in antagonizing BMP effects. miR-21 negatively regulates BMP dependent tumor suppressor genes such as *Pten*, *Pdcd4*, *Timp3* and *Tpm1* while the expression of differentiation genes *Idl*, *Id2*, *Id3* and *Msx2* are unaffected. BMP exerts its effect on keratinocytes through regulated expression of miRNAs. BMP4 dramatically inhibits miR-21 expression in keratinocytes [157].

The question is, where do the signals driving the tumors come from? What type of mutations occurs in genes and when do they happen? Are all the cells in a tumor involved in its growth equally? How are basic mechanisms of HF regeneration involved in tumorigenesis? What is the role of niche signaling in regulation of tumor growth? Can certain tumors be suppressed by turning off particular signaling pathways? Should different tumors be treated in different ways? Although there are many questions left unanswered in tumor biology, recent progress has refined knowledge concerning the role of signaling pathways involved in tumorigenesis.

## 6. Conclusions

The insight gained from the signaling pathways involved in hair follicle morphogenesis will prove helpful in the research of hair follicle abnormalities arising from signaling dysregulation. Currently, only a few attempts to modify these pathways have been performed in the treatment of abnormalities such as alopecia. Although some promising drugs have emerged with the ability to target pathway effectors, providing some success in the treatment of hair follicle abnormalities, treatment attempts remain restricted due to the potential risk of carcinogenesis. Further, more in depth research is necessary to implement the knowledge acquired from these signaling pathways in stem cell based therapy.
